# Beyond Borders: *Dirofilaria immitis* Infection in Dogs Spreads to Previously Non-Enzootic Areas in Greece—A Serological Survey

**DOI:** 10.3390/vetsci11060255

**Published:** 2024-06-04

**Authors:** Isaia Symeonidou, Georgios Sioutas, Athanasios I. Gelasakis, Dimitra Bitchava, Eleni Kanaki, Elias Papadopoulos

**Affiliations:** 1 Laboratory of Parasitology and Parasitic Diseases, School of Veterinary Medicine, Faculty of Health Sciences, Aristotle University of Thessaloniki, 54124 Thessaloniki, Greece; isaia@vet.auth.gr (I.S.); gsioutas@vet.auth.gr (G.S.); 2 Laboratory of Anatomy and Physiology of Farm Animals, Department of Animal Science, Agricultural University of Athens, 11855 Iera Odos, Greece; gelasakis@aua.gr; 3 Vet in Progress Plus, Veterinary Laboratories, Agia Paraskevi, 15343 Attiki, Greece; information@vetinprogress.gr; 4 Ceva Hellas, Makariou 34, 16341 Ilioupoli Attiki, Greece; eleni.kanaki@ceva.com

**Keywords:** canine heartworm disease, *Dirofilaria immitis*, Greece, prevalence, epizootiology

## Abstract

**Simple Summary:**

*Dirofilaria immitis* is a very important parasite of dogs that is transmitted by mosquitoes. The adult parasites live in the dog’s heart and can cause severe health problems, such as heart failure and even death. Today, it is well known that in the northern areas of Greece, a country in the Mediterranean basin, the percentages of infected dogs are high, while in the southern areas there are few reports of infected dogs. The aim of this study was to address the question of whether this infection is spreading in the southern parts of Greece as well. So, we examined 1528 blood samples from dogs derived from the southern regions and compared our results to the rate of infection recorded before 2022 in these parts of the country. Indeed, the results showed that approximately 1 out of 10 dogs was infected and, most impressively, the number of infected dogs has increased almost four times since 2022. This knowledge is very important for practicing veterinarians and pet owners in order to perform routine diagnostic tests and apply control measures.

**Abstract:**

Although *Dirofilaria immitis* in dogs is considered enzootic in northern Greece, the available data on the occurrence of infection in southern parts of the country demonstrate its scarcity. The aim of this study was to update the current knowledge on *D. immitis* infection in dogs in areas of Greece previously considered non-enzootic (Central Greece, Attica, Peloponnese, North Aegean, South Aegean, Crete and the Ionian islands). In total, 1528 dog blood samples were collected from the aforementioned areas and examined by Dirochek^®^ ELISA (Synbiotics). Additionally, data published until 2022 on the prevalence of infection in these areas were compared to the data of this study. The ‘Wilson’ Score interval method (Epitools) was employed. Overall, 10.8% of dogs were positive for *D. immitis*. In detail, the prevalence was 21.7, 13.7, 10.7, 5.4, 4.7, 6.2 and 17.0% for *D. immitis*, in Central Greece, Attica, Peloponnese, North Aegean, South Aegean, Crete and the Ionian islands, respectively. Infection with *D. immitis* is recorded for the first time in Crete. The probability of a dog becoming infected has increased 4.1 times since 2022 in previously non-enzootic areas. This study denotes the spread of *D. immitis* and highlights the necessity for preventive measures.

## 1. Introduction

Dirofilariosis is an important vector-borne parasitic disease of animals and humans. The causative agents are the filarial nematodes of the genus *Dirofilaria* (Spirurida: Onchocercidae) transmitted to the vertebrate hosts mainly via the bite of infected female mosquitoes [1,2]. In dogs, infections are caused primarily by *Dirofilaria immitis* and *Dirofilaria repens*. *Dirofilaria immitis*, often referred to as heartworm, is a cosmopolitan parasite of dogs and the most widespread species of the genus today [3]. The adults reside in the pulmonary arterial system of the infected host and migrate to the heart’s right ventricle [4]. If the infection is left untreated, then secondary congestive heart failure occurs, eventually inducing the death of the animal [5]. Dogs are considered the main domestic reservoir for *D. immitis,* although infection has been documented in various wild mammals [6]. Moreover, *D. immitis* is relevant to public health since it has a zoonotic potential and has been linked to pneumonic dirofilariosis in humans [7,8,9].

*Dirofilaria immitis* has an indirect life cycle where culicid mosquitoes of the *Aedes* and *Culex* genera are competent vectors. In particular, *Culex pipiens* and *Aedes albopictus* are the dominant vectors of this filaria [2,10]. Mosquitoes become infected during blood feed from a microfilaraemic host, and inside them, the first stage larvae (L1) moults into L2 and later into L3 [1]. The vectors inoculate the third-stage larvae in a new host during a bloodmeal. In the definitive host, the larvae mature within a few months while migrating first in the subcutaneous tissue (L4) and then in the adipose or skeletal muscle tissue (L5 immature adults) until they enter the blood steam and eventually reside as adults in the pulmonary arteries and the heart’s right ventricle. Finally, the female adults produce microfilariae L1 that are released in the bloodstream and can be ingested by mosquitoes in order to complete the life cycle [4,11].

It is well established that Greece is enzootic for canine cardiopulmonary dirofilariosis, with a high prevalence of infection documented in the northern parts of the country. It should be noted that no distinct border exists dividing enzootic and non-enzootic areas. However, according to the published data originating from different parts of Greece and to the veterinary perception, regions below Thessaly and Epirus and above the northern parts of Central Greece are considered the border between enzootic and non-enzootic areas [12,13]. In particular, epizootiological surveys have recorded prevalence rates ranging from 6.75% up to 68% for *D. immitis* infection in dogs living in the northern areas of Greece, i.e., Macedonia, Thrace, Epirus, and Thessaly [12,13,14,15,16,17,18,19,20]. Concerning the regions of Greece that were regarded as non-enzootic (Central Greece, Attica, Peloponnese, North Aegean, South Aegean, Crete, and the Ionian islands), the recorded prevalence rates from studies up to 2022 ranged from 0% in Crete up to 10.9% in the Ionian islands [12,13,19,21].

Interestingly, in recent years, increasing evidence has supported the notion of “spreading dirofilariosis” in Europe towards previously infection-free regions [2,22,23]. Indeed, epizootiological surveys have documented that *D. immitis* infections seem to be expanding in “clean” parts of Europe [23,24,25,26,27,28]. This spread is driven by many factors that contribute more or less to the outcome. In the same frame, veterinarians in Greece have increasingly confirmed *D. immitis* infection in areas of the country that were taken into account as non-enzootic. This information remains anecdotal and is derived from personal communication. Overall, although cases of canine dirofilariosis have been recorded in the southern parts of Greece, updated epizootiological data are missing. In addition, in view of climate change as well as the prediction developed by the Ecological Niche Modelling (ENM) that forecasted the spread of the infection towards the south of Greece, the need to update the relevant knowledge becomes even more urgent [29].

Therefore, the aim of this research was to obtain up-to-date information regarding the seroprevalence of *D. immitis* infection in parts of Greece that are currently considered non-enzootic and shed light on the actual trend of the geographical distribution of this parasite.

## 2. Materials and Methods

### 2.1. Study Design—Ethical Statement

**Figure 1:**
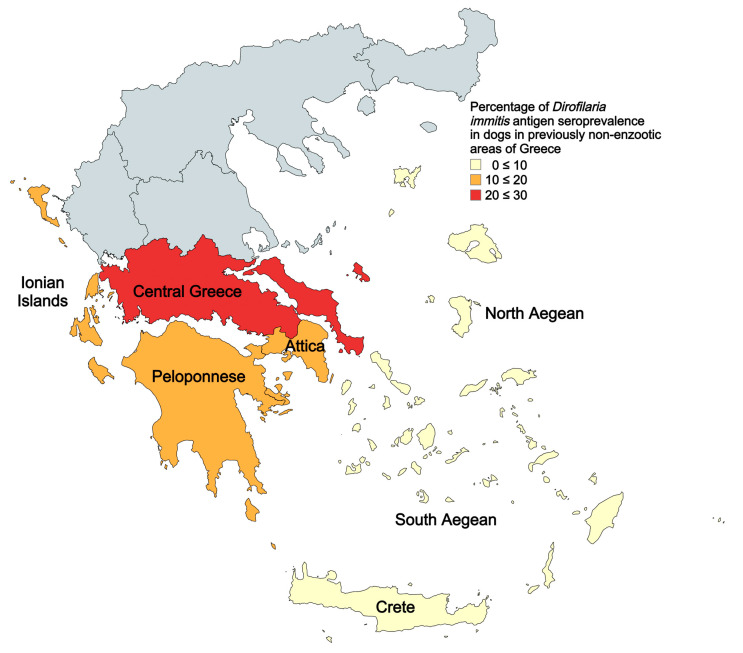
Percentage of *Dirofilaria immitis* antigen seroprevalence in dogs in 2023–2024. Areas examined represent previously (<2022) non-enzootic areas of Greece for canine heartworm disease.

To obtain a representative and coherent picture of *D. immitis* epizootiology, dogs were selected from areas that are traditionally considered non-enzootic and are distributed to the southern of Greece (Central Greece, Attica, Peloponnese, North Aegean, South Aegean, Crete, and the Ionian islands) (Figure 1). The dogs included were randomly selected; all were adults older than one year, of both sexes and of various breeds. At the time, they were not given any preventive treatment for the parasite. All animals were presented to private veterinary clinics by their owners for routine visits in 2023 and 2024. Sera samples were provided by veterinary practices during other diagnostic investigations. Moreover, all published data (including the relevant studies and their citations) regarding the prevalence of *D. immitis* infection in dogs in the areas of the country mentioned above were collected from any available source (Pubmed, Google Scholar, Scopus and Web of Science).

### 2.2. Blood Sample Collection and Serological Examination

Blood was drawn from a peripheral vein from each dog. A total of 1528 blood samples from adult dogs from the aforementioned regions of Greece were analysed (Table 1). After collection, sera were centrifugated and tested using the Dirochek® ELISA kit (Zoetis, Florham Park, NJ, USA) to detect the *D. immitis* antigen according to the manufacturer’s recommendations.

### 2.3. Statistical Analyses

Prevalence and 95% confidence intervals were estimated using the ‘Wilson’ Score interval method (Epitools) for the non-enzootic regions before 2022 and for the years 2023–2024, while the odds ratio and 95% confidence interval were estimated between them.

## 3. Results

The mean seroprevalence in 2023–2024 was 10.8% (165/1528), and the seroprevalence for each region was the following: Central Greece: 21.7% (23/106), Attica: 13.7% (55/401), Peloponnese: 10.7% (52/487), North Aegean: 5.4% (3/56), South Aegean: 4.7% (12/258), Crete: 6.2% (10/161), and Ionian islands: 17.0% (10/59) (Table 1). The seroprevalence per region is also illustrated in Figure 1.

**Figure 2:**
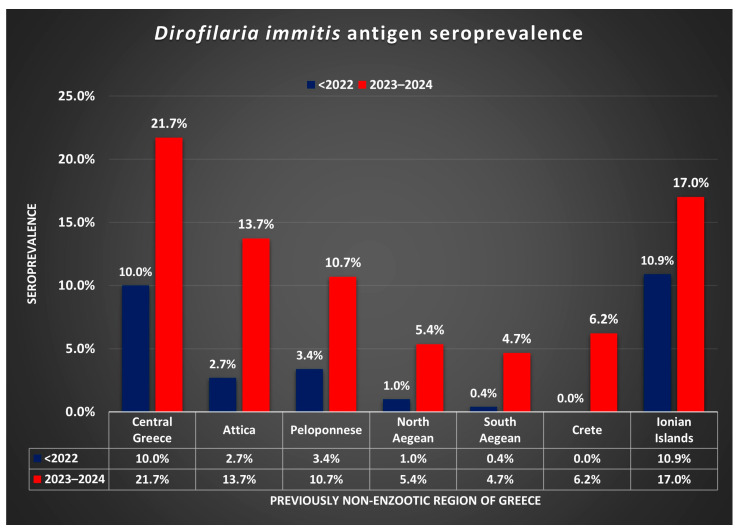
*Dirofilaria immitis* antigen seroprevalence before 2022 and in 2023–2024, in regions of Greece previously considered (<2022) non-enzootic.

Moreover, the difference in seroprevalence before 2022 and in 2023–2024 in the regions examined is illustrated in Figure 2. The likelihood of *D. immitis* infection was increased by circa 4.1 times (95% CI 3.1 to 5.4; *p* < 0.0001) in non-enzootic areas for 2023–2024 compared to before 2022.

## 4. Discussion

Until the end of the twentieth century, canine dirofilariosis caused by *D. immitis* was predominantly recorded in southern European countries, i.e., Greece, Italy, Spain, Portugal, France and Turkey. Furthermore, some sporadic cases were reported in eastern countries of Europe. Nevertheless, central and northern Europe were considered historically non-enzootic [24,25]. Twenty years later, canine cardiopulmonary dirofilariosis continues to exist and spread out in South Europe, although, most interestingly, its presence has also been established in several Eastern, Central, and even North European countries. Indeed, recent epizootiological studies have documented alterations in the distribution pattern of the infection due to a significant rise in autochthonous cases in previously “clean” areas [23,24,25,26,27]. Interestingly, in a similar way, infection with *D. repens*, i.e., the other *Dirofilaria* spp. infecting dogs, is expanding in Europe, even as far east into European Russia and as north as Finland, making climate change’s effect on the vectors more transparent [31,32]. 

Regarding Greece, it is traditionally thought as enzootic because of a Mediterranean climate where transmission is sustained for many months of the year. Several studies have demonstrated widespread infection, mainly in the country’s northern regions [12,13]. This distribution pattern, where most cases are recorded in the northern part of the country, has also been observed in neighbouring countries such as Italy [2].

In more detail, a study conducted between 1987 and 1991 in Macedonia identified microfilariae of *D. immitis* in 10% of the studied dogs [14]. In contrast, around ten years later, the prevalence rate in the same area was reported as high as 34.1% [15]. Overall, northern parts of Greece were considered enzootic for *D. immitis* since the end of the millennium [33]. In 2005, Lefkaditis and Koukeri [16] reported that dirofilariosis remains a common parasitic disease in Thessaloniki, which is located in the North, and another survey recorded a prevalence of 17.9% in dogs living on the foothills of Mt. Olympus in Northern Greece [17]. In 2016, Diakou et al. [12] sampled dogs from locations near the five major urban centres of Greece and found that 4.1% of the animals were positive for *D. immitis*, with the majority of infected dogs (18 out of the 31, 58%) living in northern regions of the country. In 2019, two studies confirmed Northern Greece as enzootic by documenting *D. immitis* infection in 25% of the examined shelter dogs [34] and 14% of dogs living in Thessaloniki [18]. Similarly, a cross-sectional serosurvey demonstrated that in northern regions, 6.75% (86/1274) of dogs were positive for the *D. immitis* antigen [19]. Interestingly, and confirming once more the hypothesis that infection is enzootic in the northern regions of Greece, Angelou et al. [13] in 2019 examined randomly selected animals from all parts of the country and recorded a 9% prevalence of infected dogs, the vast majority of them distributed only in the preceding regions. In the same context, the most up-to-date survey tested 150 dogs from Northeastern Greece (Macedonia and Thrace) and found a mean prevalence of *D. immitis* infection of 25.3%, varying from 8% to 68% in an area of Northeastern Greece (Didymoteicho) [20].

In southern parts of Greece, data are limited, and fewer studies confirm significantly lower infection rates. In Central Greece, a study recorded a 10% (19/189) prevalence of *D. immitis* infection [13]. In 2016, a survey in dogs from the Attica region failed to detect any positive cases (0/300) [12], while two studies carried out in 2019 documented prevalence rates of 3.18% (32/1006 dogs) [19] and 2.6% (7/150) [13]. In 2016, Diakou et al. [12] reported for the first time a 4.7% (7/150) rate of *D. immitis* infection in a municipality of Peloponnese, Achaia, while three years later, Angelou et al. [13] documented a lower percentage of infected dogs in Peloponnese (1.7%, 2/116). As regards North Aegean, Angelou et al. [13] identified 1 seropositive dog out of 54 sampled, while Diakou et al. [30] examined 41 dogs and documented only one positive in the island of Skiathos; however, it turned out this animal was imported from another area of the country. In the South Aegean, Angelou et al. [13] examined 127 dogs and identified one positive for *D. immitis*, whereas Diakou et al. [30] examined 159 dogs and found no positive dogs. In relation to the island of Crete, *D. immitis* infection in dogs seems to be absent until 2022. Three surveys in Crete confirmed the above by examining 251 dogs and recording the absence of *D. immitis* infection on this island [12,13,21]. In the Ionian islands, a 2019 study recorded a 10.9% (6/55) prevalence of infection [13].

The mean seroprevalence recorded in the present study was 10.8%. This percentage of infected dogs is relatively high for parts of the country where only sporadic cases were recorded up to 2022. Indeed, it was demonstrated that the likelihood of *D. immitis* infection was increased in all regions and, overall, by circa 4.1 times in non-enzootic areas for the years 2023–2024 compared to 2022. Moreover, this is the first time *D. immitis* seropositivity has been documented in dogs living on the island of Crete, the most southern part of the country.

Consequently, the present study confirms that *D. immitis* infection in dogs is spreading towards the south of Greece. This spreading is further supported by the fact that the previously non-enzootic areas bordering the enzootic ones present the highest number of seropositive dogs (Figure 2). In other words, there is a clear trend of the infection constantly expanding towards the southern parts of the country. Our findings are in concordance with analogous findings in Italy. In recent years, an increasing number of *D. immitis* autochthonous cases have been reported in dogs living in central and southern regions of Italy [35,36] and, most surprisingly, with hyperendemic foci of infection in the Pelagie archipelagos (58.9% prevalence) [37] and in Apulia region (44.2% prevalence) [38]. Furthermore, this study’s results support the concern of several Greek veterinarians in southern parts of the country who have occasionally confirmed such cases of canine dirofilariosis. 

Greece has been depicted as a country with a very high probability of *D. immitis* infection in two surveys conducted in Europe using Geographic Information Systems (GIS) [10,24]. A recent study employed Ecological Niche Modelling (ENM) to assess the dynamics of *D. immitis* infection in Greece [29]. This tool is useful for predicting the risk of transmission of various parasites [39] by incorporating in-model approaches’ biological variables and host characteristics and relating them to a specific infection [40]. Moreover, and most importantly, data obtained from the ENM tools can be utilised to conclude the pattern of infection in regions that are yet non-enzootic, and surveillance data are lacking [29]. For the case of Greece, several bioclimatic parameters and previously published records on the mosquito populations in the country, especially the dominant species in Greece, *Cx. pipiens* [39], were included in the model’s algorithm. Overall, this model predicted that the risk of *D. immitis* infection is high in regions bordering the main river basins as well as in coastal areas, irrigated areas, and islands. On the contrary, the possibility of infection was forecasted as low in areas with higher altitudes inland. Furthermore, this study generated a map of Greece where Central Macedonia, Attica, the South Aegean islands, and the island of Crete were found to have the most suitable habitat for *Cx. pipiens* and, thus, were characterised as high-risk areas. The areas mentioned above are territories with low mountains exposed to the Aegean Sea [29]. 

The change in *D. immitis* infection distribution patterns is a multifactorial process and can be attributed to several causes that have not yet been clarified. However, some factors have been established to favour the transmission of the infection. First and foremost, climate change, which has resulted in increased warm weather periods during the year, represents a major determinant for the survival of mosquitoes [10,22,41]. Indeed, arthropod vectors are susceptible to climate change since the latter drastically influences all the environmental variables that comprise their habitat, e.g., humidity, temperature, rainfall, vegetation index and others [42]. Today, our understanding of how all these parameters drive the occurrence and distribution of *D. immitis* infection in dogs is not thorough, and additional research based on GIS modelling is required [24]. However, it is a common understanding that global warming contributes to the increasing abundance of mosquitoes [43] and, therefore, to the spread of infectious mosquito-borne diseases in areas previously known as “clean” [22,44]. Moreover, the rise in temperature influences the development of the *D. immitis* larvae inside the vectors by abbreviating their moulting period [32,45].

Another crucial factor associated with the spreading of vector-borne diseases, such as *D. immitis* infections, is the travelling of pets across Greece and Europe. Since the Pet Travel Scheme launch in 2000, there has been an elevated interest due to the lessening of restrictions on the movement of pets between countries of the continent [46]. This concern was proven justified since it was later denoted beyond doubt that the travel of infected, microfilaraemic dogs from enzootic regions across Europe contributed to the spread of infection [10,22]. 

Furthermore, another factor favouring the distribution of filarial infections is the abundance of wild reservoirs [43]. The role of wild animals in the epizootiology of dirofilariosis should not be underestimated. It has been proven that *D. immitis* infects many wild mammalian species, such as golden jackals (*Canis aureus*), wolves (*Canis lupus*), coyotes (*Canis latrans*), red foxes (*Vulpes vulpes*), European wildcats (*Felis silvestris silvestris*), black bears (*Ursus americanus*), brown bears (*Ursus arctos*) and European badgers (*Meles meles*) [47,48]. In addition, cardiopulmonary dirofilariosis has been identified as one of the leading causes of death in wildlife animals [49]. However, until now, there is a lack of data regarding the exact epizootiological role of wildlife animals in transmitting *D. immitis* since their role as definitive hosts for this parasite has not been adequately investigated [47]. In Greece, there are limited studies, and the presence of the parasite has been documented in a golden jackal [50], a brown bear [51], and most recently in two European badgers [48]. In the latter study, the presence of microfilariae in both badgers was detected, thus corroborating this wildlife species as a competent host. This later finding is of particular significance since both animals lived in Crete, representing autochthonous cases. Although it was not previously detected, we cannot exclude the possibility that the parasite circulated before 2022 among the canine population, i.e., in stray dogs. Nowadays, the percentage of infected dogs is increasing (6.2%), as predicted by the ENM. To exacerbate the fact that badgers contribute to the maintenance of infection, it should be pointed out that although badgers thrive in Crete, other wild animals, including golden jackals, grey wolves, red foxes and brown bears, which serve as competent hosts for the parasite, are absent in this island. 

As stated above, in Italy, a similar trend of new cases arising in the country’s southern regions was recorded [2]. Researchers attributed this spread of infection to the fact that in non-enzootic areas, chemoprophylaxis for *D. immitis* is not performed routinely by practitioners and owners [35]. Likewise, it has been speculated that the lack of chemoprophylaxis as well as chemotreatment in dogs in the southern regions of the same country is the cause of the impressively high infection rates recorded in these areas [37,38]. We could safely assume that this is also a contributing factor in the case of Greece.

Monitoring the distribution of *D. immitis* infection is essential for guarding animal and human health. In dogs, infection may lead to the development of cardiopulmonary disease, which is fatal if not treated [5]. As mentioned, both *Dirofilaria* species affecting dogs are zoonotic, and human infections, primarily due to *D. repens*, are increasing in Europe [7,52]. Noteworthily, a strong association has been established linking cases in humans with a high prevalence rate of subcutaneous dirofilariosis in dogs, thus depicting the zoonotic nature of this infection [53]. Concerning *D. immitis*, humans are not regarded as optimal hosts, and thus, the migrating larvae eventually die, producing infracts usually in the lung vessels [8,9]. These infracts are referred to as “coin lesions” in X-rays. Furthermore, infected individuals with *D. immitis* exhibit mild eosinophilia and systemic clinical signs such as pyrexia and respiratory distress. Humans infected with *D. immitis* can also develop nodules in certain cases [54].

In this study, the Dirochek^®^ ELISA kit was employed. This commercially available, easy-to-perform test detects the female adult *D. immitis* antigen. It consists of a microwell format heartworm antigen plate assay that employs enzyme-linked immunosorbent assay (ELISA) technology. This canine heartworm antigen kit is highly specific and sensitive. When evaluated in a blinded study, it was significantly more sensitive than other antigen test kits [55]. Since results are obtained in 15 min, this test is commonly used where there is a need for high volumes of testing, as in referral laboratories or sero-epizootiological studies. Regarding the effect of heat treatment on the accuracy of the DiroCHEK^®^ Antigen Test, a study proved that this assay was 100% accurate in detecting the presence of live adult heartworms without needing heat treatment [56]. Concerning the limitations of the present study, one could speculate that the actual prevalence of *D. immitis* infection is overestimated since certain cases of seropositive dogs may be animals not autochonous, as they might have been infected during travelling to other enzootic areas. On the other hand, although no targeted prevention for *D. immitis* was administered in the examined animals, the possibility of treating with macrocyclic lactones for any other reason could have resulted in protection and therefore seronegativity; thus, any underestimation of the prevalence cannot also be excluded.

The epizootiological surveillance of *D. immitis* infection in dogs should be an ongoing task. This will help practitioners to routinely diagnose the infection even in areas that are considered non-enzootic to date. In addition, it will help to increase awareness among pet owners. It is essential to educate pet owners during routine visits on the importance of systematic prevention, the dangers associated with travel, and the benefits of periodic testing of their dogs. The above will provide the basis for taking more optimal prophylactic measures so as to safeguard animals and, given the zoonotic potential of the parasite, humans. Prevention will interrupt the spread of canine cardiopulmonary dirofilariosis since microfilariaemic dogs, especially those without clinical signs and treatment, are reservoirs. Further future studies investigating the occurrence of dirofilariosis in animals, both domestic and wild, and humans, as well as the dynamics of its vectors, should be carried out, particularly in regions of Europe that are referred to as non-enzootic.

## 5. Conclusions

The current study confirms that *D. immitis* infection in dogs is spreading towards the south of Greece since canine seroprevalence rates have increased in all Greek regions previously regarded as non-enzootic. Compared to before 2022, the likelihood of a dog becoming infected with the parasite in the above-mentioned areas has increased 4.1 times. Furthermore, infection with *D. immitis* is recorded for the first time in dogs on the island of Crete, the most southern part of the country. Therefore, it is necessary to increase awareness among veterinary practitioners and pet owners nowadays and implement effective prevention measures in all areas of Greece to minimise the risk of infection and thus protect animal and human health.

## Figures and Tables

**Table 1:** Prevalence and 95% confidence intervals for the non-enzootic regions before 2022 and for 2023–2024.

Regions	Tested Dogs	Positive Dogs	Prevalence of *D. immitis* (%)	95% Confidence Interval of Prevalence		
				Lower (%)	Upper (%)	
2023–2024						
Central Greece	106	23	21.7	14.9	30.5	
Attica	401	55	13.7	10.7	17.4	
Peloponnese	487	52	10.7	8.2	13.7	
North Aegean islands	56	3	5.4	1.8	14.6	
South Aegean islands	258	12	4.7	2.7	8.0	
Crete	161	10	6.2	3.4	11.1	
Ionian islands	59	10	17.0	9.5	28.5	
Total	1528	165	10.8	9.3	12.5	
<2022						References
Central Greece	189	19	10.1	6.5	15.2	[13]
Attica	1456	39	2.7	2.0	3.6	[12,13,19]
Peloponnese	266	9	3.4	1.8	6.3	[12,13]
North Aegean islands	95	1	1.1	0.2	5.7	[13,30]
South Aegean islands	286	1	0.4	0.0	2.0	[13,30]
Crete	251	0	0.0	0.0	1.5	[12,13,21]
Ionian islands	55	6	10.9	5.1	21.8	[13]
Total	2598	75	2.9	2.3	3.6	

## Data Availability

The original contributions presented in the study are included in the article, further inquiries can be directed to the corresponding author.
